# Regulation of Lymphatic Function in Obesity

**DOI:** 10.3389/fphys.2020.00459

**Published:** 2020-05-15

**Authors:** Raghu P. Kataru, Hyeong Ju Park, Jung Eun Baik, Claire Li, Jinyeon Shin, Babak J. Mehrara

**Affiliations:** Plastic and Reconstructive Surgery Service, Department of Surgery, Memorial Sloan Kettering Cancer Center, New York, NY, United States

**Keywords:** lymphatic vessels, lymphatic function, obesity, inflammation, metabolic syndrome

## Abstract

The lymphatic system has many functions, including macromolecules transport, fat absorption, regulation and modulation of adaptive immune responses, clearance of inflammatory cytokines, and cholesterol metabolism. Thus, it is evident that lymphatic function can play a key role in the regulation of a wide array of biologic phenomenon, and that physiologic changes that alter lymphatic function may have profound pathologic effects. Recent studies have shown that obesity can markedly impair lymphatic function. Obesity-induced pathologic changes in the lymphatic system result, at least in part, from the accumulation of inflammatory cells around lymphatic vessel leading to impaired lymphatic collecting vessel pumping capacity, leaky initial and collecting lymphatics, alterations in lymphatic endothelial cell (LEC) gene expression, and degradation of junctional proteins. These changes are important since impaired lymphatic function in obesity may contribute to the pathology of obesity in other organ systems in a feed-forward manner by increasing low-grade tissue inflammation and the accumulation of inflammatory cytokines. More importantly, recent studies have suggested that interventions that inhibit inflammatory responses, either pharmacologically or by lifestyle modifications such as aerobic exercise and weight loss, improve lymphatic function and metabolic parameters in obese mice. The purpose of this review is to summarize the pathologic effects of obesity on the lymphatic system, the cellular mechanisms that regulate these responses, the effects of impaired lymphatic function on metabolic syndrome in obesity, and the interventions that may improve lymphatic function in obesity.

## Introduction

### Lymphatic Anatomy and Physiology

The lymphatics are a component of the vascular system and are present only in vertebrate animals. The lymphatic system is comprised of open-ended initial or capillary lymphatic vessels that drain successively into larger collecting lymphatics. Capillary lymphatic vessels comprise of single layer of overlapping oak-leaf shaped lymphatic endothelial cells (LECs) with interendothelial gaps, discontinuous button-like junctions that enable interstitial fluid absorption and leukocyte entry. Collecting lymphatic vessels (CLVs) are surrounded by basement membrane, covered with lymphatic muscle cells (LMC) and contain bileaflet intraluminal valves that prevent lymph back flow. Unlike capillary lymphatic vessels CLVs exhibit zipper-like interendothelial junctions to prevent lymph leakage. The contractile activity of LMCs of CLVs help actively pump interstitial fluid and drain into lymph nodes that then filter the fluid for antigens and bacteria (Alitalo et al., 2005; Baluk et al., 2007; Tammela and Alitalo, 2010; Wang et al., 2017). Efferent lymphatics drain from lymph nodes into larger CLVs that eventually return the interstitial fluid back into the blood circulation (Petrova and Koh, 2018; Liu and Oliver, 2019). In this manner, the lymphatic system plays a key role in regulating fluid homeostasis, immune cell migration and antigen presentation, resolution of inflammatory responses, and regulation of peripheral tolerance to self-antigens (Angeli and Randolph, 2006; Kim et al., 2012; Betterman and Harvey, 2016; Randolph et al., 2017).

The earliest observation of lymphatic vessels was made based on the ability of intestinal lymphatic vessels to uptake lipids after a fat-rich meal. In 300 B.C., ancient Greeks observed milk-filled intestinal lymphatics and provided the first anatomic description of the lymphatic system (Lord, 1968). In the 17th Century, Gaspero Aselli identified these milk-filled vessels as components of the vascular system (Asellius, 1627). In the last few decades, the discovery of lymphatic-specific markers helped to differentiate lymphatic vessels from the blood vasculature, and to identify the vital role of the lymphatic system in absorption of dietary fat and cholesterol transport. The intestinal lymphatics are known as lacteals and are located centrally within each intestinal villus. Dietary fats are converted by enterocytes into triglyceride-rich lipoproteins enveloped by proteins and cholesterol, which are called chylomicrons, and absorbed and transported by the intestinal lacteals by both passive and active contractile mechanisms (Choe et al., 2015; Escobedo and Oliver, 2017; Suh et al., 2018; Cifarelli and Eichmann, 2019; Hokkanen et al., 2019). Intestinal lacteals can also absorb fat-soluble vitamins. Once taken up by the lacteals, chylomicrons are transported by lymphatic vessels and drained into the systemic venous circulation, bypassing the portal venous system. This is important since bypassing the liver decreases first-pass metabolism of dietary compounds and is, as a result, an active area of research in the pharmaceutical industry.

Peripheral lymphatics also play a role in cholesterol transport by a mechanism known as reverse cholesterol transport (RCT). In this process, cholesterol molecules deposited in peripheral tissues are released by cells facilitated by lipid free apolipoprotein A1 (APOA1) or lipidated high-density lipoprotein (HDL). This extracellular cholesterol is transported by the lymphatic system back to the blood stream and liver, where they are excreted or processed. This mechanism is thought to play an important role in regulating pathological processes such as atherosclerosis. Thus, understanding how the lymphatics regulate this process may facilitate development of novel therapies for treatment of cardiovascular diseases (Lim et al., 2013; Martel et al., 2013; Huang et al., 2015).

Defects in lymphatic development or function resulting from a variety of genetic defects result in adipose deposition, changes in cholesterol metabolism, metabolic changes, and, in some cases, adult-onset obesity depending on the degree of lymphatic structural and functional abnormality (Karkkainen et al., 2001; Gale et al., 2002; Harvey et al., 2005; Dellinger et al., 2007, 2008). These findings suggest that the lymphatic system and obesity are related, and that this interaction is bi-directional.

Previous reviews covered several aspects of obesity and its implication on lymphatic vessels in relation to different pathologies like lymphedema and metabolic disorders (Escobedo and Oliver, 2017; Jiang et al., 2019; Ho and Srinivasan, 2020). This article emphasizes on the bidirectional interaction between obesity and lymphatic function. We highlighted how inflammatory pathologic changes of obesity in the form of peri-lymphatic inflammation adversely affect lymphatic structure, function and how these changes predispose obese individuals to lymphedema and metabolic complications. We also discussed how life style and pharmacological interventions mitigate inflammation to improve lymphatic function in obesity.

## Obesity Causes Lymphatic Dysfunction and Increases Risk of Lymphedema

The etiology of obesity is, in most cases, related to excess calorie consumption and limited caloric expenditure, however, in rare cases, obesity may also be caused by genetic abnormalities. Obesity is a systemic disease affecting virtually every organ system and increases the risk of developing metabolic syndrome and a variety of malignancies (Barton et al., 2012; Barton, 2013; Flegal et al., 2013; Segula, 2014; Kim et al., 2016).

Recent studies have also shown that obesity and adipose related disorders have significant negative effects on the lymphatic system. For example, clinical studies have demonstrated that obese individuals have impaired subcutaneous clearance of macromolecules (Arngrim et al., 2013). Patients with Dercum’s disease, a poorly understood disorder characterized by accumulation of subcutaneous adipose tissue and painful lipomas, have structural and functional lymphatic defects (Rasmussen et al., 2014). Lipedema, another subcutaneous adipose tissue disorder with excessive fat deposition under skin, exhibits lymphatic vessel microaneurysms causing leakage of lipids from damaged and leaky lymphatic vessels causing excessive adipogenesis (Stallworth et al., 1974; Herbst, 2000; Amann-Vesti et al., 2001; Al-Ghadban et al., 2019). Reports indicate that lymphatic transport defects are more prominent in late stage lipedema compared to early stages (Gould et al., 2020). In similar lines, research indicates that lymphedema a chronic, morbid disease characterized by tissue swelling, accumulation of fluids, fibro-adipose tissue spontaneously develops in severely obese individuals. Early research showed the occurrence of massive localized lymphedema in morbidly obese individuals (Farshid and Weiss, 1998). Furthermore, several studies show that obesity is a well-recognized risk factor and body mass index (BMI) has a direct correlation with development of spontaneous as well as secondary lymphedema following lymph node dissection for cancer treatment (Swenson et al., 2009; Greene et al., 2012; Greene and Maclellan, 2013; Mehrara and Greene, 2014; Maclellan et al., 2017). This is supported and likely related to the fact that baseline lymphatic function is a predictor of lymphedema development and severity of disease (Hespe et al., 2019). As a result, obese patients are 2- to 3-fold more likely to develop lymphedema after surgery (Clark et al., 2005; Arrault and Vignes, 2006; Helyer et al., 2010). Even postoperative weight gain (and, presumably, the altered lymphatic function that ensues) increases the risk of lymphedema development (McLaughlin et al., 2008). Finally, recent studies have demonstrated that weight loss is an effective means of treating lymphedema and decreasing its symptoms (Shaw et al., 2007; Ridner et al., 2011), however, not in the case of dercum’s disease, in which lipomas still exist despite weight loss (Herbst and Asare-Bediako, 2007).

### Animal Models Reveal That Obesity Results in Structural and Functional Changes in the Lymphatic System

Animal models of obesity have been used by several groups to study the cellular mechanisms that regulate development of obesity-induced lymphatic dysfunction. A commonly used mouse model is high fat diet (HFD)-induced obesity. This model is thought to reflect changes that occur in spontaneous obesity (in contrast to genetically induced models of obesity) and requires feeding adult animals a diet in which 60–70% of the total calories are derived from fat. Typically, adult male C57BL/6J mice are maintained on an HFD for 10–12 weeks and are compared to littermates fed a normal chow diet (30% calories from fat) (Savetsky et al., 2014). Male mice are much more prone to HFD-induced obesity and are used by most researchers. Female mice also gain weight following prolonged exposure to HFD, but to a lesser extent than male mice with over all less body fat levels; however, this process can be accelerated by ovariectomy. Ovariectomized female mice gained weight similar to male mice indicating that ovarian hormones might have a protective effect on female mice in weight gain (Hong et al., 2009). Report indicates that, 10–12 weeks of HFD feeding regime showed higher the blood glucose, insulin, liver enzymes, plasma lipids, cholesterol, adipose tissue macrophages, and circulating leukocytes in male mice compared to female (Singer et al., 2015; Ingvorsen et al., 2017). However, long term HFD regime (24 weeks) results in no differences between male and female in any of the parameters mentioned above indicating duration of high fat feeding is critical in exhibition of sexual dimorphism in HFD induced obesity (Bruder-Nascimento et al., 2017). Most common mice strains like C57BL/6J, FVB/N, 129 × 1, DBA/2 are prone for obesity upon HFD feeding but BALB/c mice strain is found to show certain degree of resistance to obesity even after HFD feeding (Montgomery et al., 2013). Other studies have used genetically induced models of obesity such as leptin deficient mice (Zhang et al., 1994). These animals become obese spontaneously, regardless of the diet, but are less representative of obesity clinically, since only a small percentage of obese patients have genetic abnormalities.

Studies in HFD-induced obese mice have consistently shown that obese animals have decreased lymphatic density in subcutaneous tissues, reduced LEC proliferation, increased lymphatic leakiness of both initial and collecting lymphatics, decreased collecting-vessel pumping capacity, and impaired clearance of macromolecules (Weitman et al., 2013; Blum et al., 2014; Garcia Nores et al., 2016; Torrisi et al., 2016). Not surprisingly, obese mice have significantly decreased trafficking of dendritic cells (DCs) from tissues to regional lymph nodes, have structurally abnormal and dilated lymphatic vessels, and have lymph node abnormalities consisting of decreased lymph node size, irregular architecture and loss of chemokine gradients (Weitman et al., 2013; Blum et al., 2014). Reports also indicated that obese mice lymph nodes showed loss of follicular pattern, T-cell zones and impaired *ex vivo* T-cell recall ability after *in vivo* sensitization with 1-fluoro-2,4-dinitrobenzene (DNFB) (Savetsky et al., 2015a; Hespe et al., 2016).

### Obese Animals Have Decreased Expression of Lymphatic Genes by Isolated LECs

How does obesity cause lymphatic vessel abnormalities and decrease LEC proliferation? Several lines of evidence suggest that HFD-induced obesity results in marked alterations in LEC gene expression. Using fluorescence-activated cell sorting to isolate LECs from dermal samples, studies in our lab have shown that obese mice have downregulated expression of LEC genes including lymphatic vessel endothelial hyaluronan receptor 1 (LYVE-1), prospero-related homeobox 1 (PROX-1), podoplanin, and vascular endothelial growth factor receptor 3 (VEGFR-3) (Garcia Nores et al., 2016; Hespe et al., 2016; Nitti et al., 2016). It is well known that, the transcription factor PROX-1 is the master regulator of LEC differentiation and VEGFR-3 is the main receptor for vascular endothelial growth factor C (VEGF-C) and vascular endothelial growth factor D (VEGF-D), the most important lymphangiogenic growth factors. Binding of VEGFR-3 by VEGF-C/D results in activation of downstream signaling pathways that are key regulators of LEC proliferation, differentiation, and protection from apoptosis. Thus, decreases in VEGFR-3 transcription and cell surface expression in LECs of obese animals are likely important mechanisms by which obesity decreases lymphatic function. This hypothesis is supported by the fact that expression of VEGF-C is increased in the tissues and serum of obese mice and patients (Silha et al., 2005; Garcia Nores et al., 2016; Zafar et al., 2018). This increased expression of the receptor ligand VEGF-C may represent a homeostatic effect to maintain VEGFR-3 activation. Such a homeostatic regulation of insulin secretion by pancreatic β-cells in response to decreased insulin receptors or vice versa is observed in diabetic individuals (Zick, 2005; Rhodes et al., 2013). Additional research is clearly needed to determine how obesity modulates the VEGFR-3/VEGF-C signaling axis, and how these changes translate to lymphatic abnormalities *in vivo*.

### Obesity-Induced Inflammation and Lymphatic Function

Chronic, low-grade inflammation is a key cellular mechanism that regulates the pathophysiology of obesity in a variety of organ systems. This phenomenon also appears to hold true for the effects of obesity on the lymphatic system. For example, several studies from our lab have shown that HFD-induced obesity results in peri-lymphatic accumulation of leukocytes (CD45^+^ cells) (Garcia Nores et al., 2016; Torrisi et al., 2016). Interestingly, we found that not only do obese mice have increased generalized low-grade inflammation in their subcutaneous fat, but that these inflammatory cells tended to accumulate around lymphatic vessels (both capillary and collecting lymphatics) (Savetsky et al., 2015a; Torrisi et al., 2016; Ariyagunarajah and Chen, 2017). This peri-lymphatic inflammatory response was mixed in nature consisting of CD11b^+^/inducible nitric oxide synthase (iNOS)^+^ macrophages and CD4^+^ T-lymphocytes. Although the mechanisms regulating the peri-lymphatic clustering of inflammatory cells remain unknown, some authors have shown changes in expression patterns of chemokines such as C-C motif chemokine ligand 21 (CCL21) (Weitman et al., 2013). Gradients of CCL21 regulate inflammatory cell migration into lymphatics, suggesting that the loss of these gradients in obese mice may result in trapping of inflammatory cells in tissues (Johnson and Jackson, 2010; Tal et al., 2011; Russo et al., 2016). In addition, CLV permeability is reported to facilitate peri-nodal adipose tissue inflammation. CLVs leak lymph born antigens enabling sampling by adipose tissue DCs and T cell recruitment during recall responses (Kuan et al., 2015). Furthermore, leaky CLVs facilitate severe adipose deposition, chronic inflammation and tertiary lymphoid organ formation in mesentery of Crohn’s disease patients aggravating the disease pathology (Randolph et al., 2016). Another mechanism that may contribute to white adipose tissue inflammation is increased expression of growth factors such as vascular endothelial growth factor A (VEGF-A) and VEGF-C in obese individuals (Miyazawa-Hoshimoto et al., 2003; Tinahones et al., 2012; Zafar et al., 2018). VEGF-C is highly chemotactic for myeloid-derived cells such as macrophages (Skobe et al., 2001; Nurmi et al., 2015; Shew et al., 2018). High levels of VEGF-C can also increase blood and lymphatic leakiness, macrophage infiltration and may impair lymphatic function in a feed forward manner (Jeon et al., 2008; Gousopoulos et al., 2017). An interesting study demonstrated that blockade of VEGF-C is an effective means of preventing development of insulin resistance by decreasing macrophage chemotaxis to subcutaneous tissues (Karaman et al., 2015).

A recent study reported that enhancing adipose tissue-specific lymphangiogenesis not only improves adipose tissue lymphatic function but also decreases metabolic abnormalities (Chakraborty et al., 2019). In this study, a novel mouse model of adipose tissue-specific overexpression of VEGF-D (Adipo-VD mice) was created, resulting in abundant functional lymphangiogenesis in white adipose tissue. Adipo-VD mice fed an HFD had improved glucose clearance, lower insulin levels, and reduced triglycerides from the liver as compared to controls. Functionally, these mice exhibited higher glycerol flux from adipose tissue and decreased number of macrophage-positive crown-like structures as compared to controls suggesting that these animals had improved immune cell trafficking from adipose tissue. This study therefore suggests that the effects of lymphangiogenic growth factor expression are context dependent and variable depending on the target cell (i.e., adipocyte versus inflammatory cells).

Several lines of evidence suggest that peri-lymphatic inflammatory responses play a role in the development of obesity-induced lymphatic dysfunction. For example, mice deficient in CD4^+^ cells (CD4 knockout), athymic nude mice (deficient in T cells), or Rag-1 mice (deficient in both T and B cells) have decreased peri-lymphatic inflammation after prolonged exposure to HFD and have improved lymphatic function as compared with similarly fed wild-type controls (Weitman et al., 2013; Torrisi et al., 2016). Topical application of tacrolimus, an IL-2 inhibitor that decreases T cell proliferation and differentiation, results in decreased lymphatic leakiness and improved collecting lymphatic pumping capacity in obese mice (Torrisi et al., 2016). Tacrolimus applied topically poorly absorbed into systemic circulation and the improvements in lymphatic function were only limited to the site of drug delivery, suggesting that local changes in inflammation rather than systemic effects of obesity were responsible for obesity-induced lymphatic leakiness and abnormal pumping. This hypothesis is supported by the fact that there was no difference in total body weight or adipose tissue weights in animals treated with topical tacrolimus versus controls. However, topical tacrolimus treatment significantly reduced the number of CD4^+^ T cells and related inflammation locally, correlating with the increased lymphatic function. Thus, neutralizing T cell inflammation seems to have a strong therapeutic value to enhance lymphatic function in obesity. In fact, previous research demonstrated that neutralization of CD3^+^ T cells with systemic antibody therapy decreases metabolic syndrome and insulin resistance in obese mice (Winer et al., 2009). It will be interesting to see how systemic CD3^+^ cell neutralization effects lymphatic function in obesity.

Behavioral modifications that decrease inflammation also improve lymphatic function. It is well established that lifestyle modifications such as caloric restriction or aerobic exercise training significantly decrease white adipose tissue, skeletal muscle inflammation, iNOS, and release of inflammatory cytokines (Bruun et al., 2006; Bradley et al., 2008; Kawanishi et al., 2010; Baynard et al., 2012; Kawanishi et al., 2013). These interventions also improve glucose intolerance and insulin sensitivity, decrease adipose tissue inflammation and adipokine production, and improve cardiac function (Sjostrom et al., 2000; Poirier et al., 2003; Clement et al., 2004; Sjostrom et al., 2004; Moschen et al., 2010). More recently, it has become clear that behavioral modifications also improve lymphatic function. For example, HFD-induced obese mice that underwent a 6-week course of aerobic exercise training that did not cause weight loss but did decrease white adipose tissue inflammation had significantly improved lymphatic function (decreased leakiness, improved pumping) as compared with sedentary obese controls (Hespe et al., 2016). These findings are interesting, as they suggest that lymphatic dysfunction in obesity is regulated by paracrine responses rather than direct effects of dietary toxins or physical compression of lymphatic vessels by enlarged adipocytes. In another study, caloric restriction of obese mice not only resulted in weight loss but also decreased white adipose tissue inflammation and restored lymphatic function to near normal levels (Nitti et al., 2016). This study also demonstrated a threshold effect from weight gain (weight > 40 g) beyond which lymphatic functional deficits become more pronounced and measurable. These findings are important because they suggest that obesity-induced lymphatic dysfunction is reversible (at least partially), and that behavioral modifications or pharmaceutical interventions that decrease inflammation may aid in this process.

Peri-lymphatic inflammatory cells, particularly macrophages, strongly express iNOS (Mattila et al., 2013; Xue et al., 2018). It has been reported that, collecting lymphatic pumping is regulated by gradients of NO and, under normal circumstances, NO production around lymphatic vessels is secondary to the expression of endothelial nitric oxide synthase (eNOS) (Berg and Scherer, 2005; Lahdenranta et al., 2009; Liao et al., 2011; Mauricio et al., 2013; Iantorno et al., 2014). Valvular and tubular lymphatic segments increase NOS expression during phasic contractions in turn regulate lymphatic contraction and relaxation (Bohlen et al., 2009). These findings suggest that the loss of NO gradients around collecting lymphatics due to high levels of iNOS expression by perilymphatic inflammatory cells impairs lymphatic pumping and results in collecting lymphatic dilatation (a phenotype that is commonly observed). This conclusion is supported by the fact that *in vivo* inhibition of iNOS in obese mice using a small molecule inhibitor, 1400 W, significantly improves lymphatic pumping and overall lymphatic function (Torrisi et al., 2016), and increased levels of iNOS are known to cause abnormalities in lymphatic contractile function in a variety of pathologic settings (Liao et al., 2011; Scallan et al., 2016). On the other hand, *ex vivo* experimental reports suggests basal NO causes decreased contractile function and lymph flow (Scallan and Davis, 2013).

High concentrations of NO decrease lymphatic contraction frequency and amplitude of contractions (Gashev et al., 2002; Gasheva et al., 2006, 2007). High levels of iNOS and NO may also regulate lymphatic function by other mechanisms. For example, NO is an oxygen donor, and high local concentrations of this molecule may result in generation of free oxygen and free nitrogen radicals and LECs are sensitive to free radical injury (Kasuya et al., 2014). Free radicals and iNOS itself also regulate inflammatory cell migration and may promote chronic inflammatory reactions (Liang et al., 2016). Obese iNOS knockout mice have improved metabolic parameters, decreased insulin resistance, and, most importantly, decreased tissue inflammation (Perreault and Marette, 2001). Whether or not these animals also have improved lymphatic function remains to be seen, and this is a topic of active study in our laboratory.

Inflammatory cells are also a major source of cytokines that may have important effects on LECs. For example, T cells are major sources of cytokines including interferon gamma [IFN-γ, interleukin 4 (IL-4), IL-13, and transforming growth factor beta (TGF-β)]. These cytokines have potent anti-lymphatic effects, and downregulate LEC proliferation and function *in vivo* and *in vitro* (Clavin et al., 2008; Kataru et al., 2011; Savetsky et al., 2015b; Shin et al., 2015). Thus, subcutaneous tissue inflammatory responses may directly inhibit lymphangiogenesis and lymphatic function by increasing the expression of anti-lymphangiogenic cytokines.

### Is It Obesity or HFD?

Is it possible that some of the lymphatic abnormalities noted in HFD-induced obese mice may be related to toxic compounds in their diet or perhaps generated by high levels of dietary fat? To study this question, we compared lymphatic function in genetically obesity-resistant [myostatin (MSTN) knockout and BALB/c mice] and obesity-prone (C57BL/6J) mice that were fed on a HFD for prolonged periods of time (Garcia Nores et al., 2016). Not surprisingly, obesity-prone mice fed an HFD progressively increased in weight and became obese after 12–14 weeks of HFD feeds. In contrast, obesity-resistant mice gained only modest amounts of weight and did not become obese. Obesity-prone mice fed an HFD had severely abnormal lymphatic function, leaky lymphatics, impaired collecting vessel pumping, and decreased LEC mRNA expression of lymphatic-specific markers, including VEGFR-3. These mice also had abnormal metabolic parameters, including hyperinsulinemia and increased serum leptin as well as accumulation of crown-like structures (macrophages engulfing enlarged adipocytes) (Boi et al., 2016). In contrast, obesity-resistant mice fed an HFD had essentially normal lymphatic function, preserved lymphatic architecture/anatomy, and normal metabolic parameters. More importantly, these mice had limited white adipose tissue inflammation and very few peri-lymphatic inflammatory cells. Taken together, these findings suggest that obesity and inflammation are both important regulators of lymphatic dysfunction, and that dietary modifications alone are insufficient to induce this effect.

### Role of Adipokines and Free Fatty Acids

In addition to inflammation, obesity induces the expression of a variety of adipokines and accumulation of free fatty acids (FFAs) in adipose tissues, and several studies have suggested that these products can negatively impact lymphatic function. For example, high levels of leptin, as noted in obese individuals, strongly inhibits LEC tubule formation/proliferation, and leptin treatment results in aberrant morphological changes in human lymphatic ducts (Leal-Cerro et al., 1996; Rose et al., 2002; Sato et al., 2016). Adiponectin, a cytokine with a positive effect on obesity, is reported to be found in low levels in obese patients (Shibata et al., 2009; Nigro et al., 2014). Interestingly, adiponectin is found to have a protective effect on lymphatic vessels by LEC differentiation and viability. This is also supported by the finding that adiponectin-knockout mice show reduced lymphangiogenesis and exacerbated lymphedema phenotype (Shimizu et al., 2013).

FFAs are abundant in tissues of obese subjects, and their role in insulin resistance and inflammation in target tissues is well studied (Boden, 2008). Reports indicate that FFAs, especially stearic acid, induced significantly more blood endothelial cell apoptosis and necrosis (Harvey et al., 2010). Treatment with oleic acid caused hyperpermeability of LECs *in vitro* (Sawane et al., 2013). Recent studies on cultured LECs show that some FFAs are highly toxic to LECs even in low concentrations by causing dose-dependent increase in apoptosis, decreased proliferation and downregulation of PROX-1 and VEGFR-3 expression, (Garcia Nores et al., 2016). These reports indicate that increased tissue concentration of FFAs in obese subjects can directly injure the lymphatic endothelium. In addition to direct toxic effects on LECs, FFAs may interact with other molecules that regulate lymphatic vessel stability. For example, apelin, a well-known endogenous ligand orphan G protein-coupled receptor (APJ), is an important regulator of lymphatic vessel stability and integrity (Sawane et al., 2011). Apelin-knockout mice exhibit hyperpermeability and abnormal skin lymphatic function under HFD-fed conditions, due to elevated plasma FFAs and their negative effects on LECs. The lymphatic defective phenotype in Apelin-knockout mice is reversed in Apelin-transgenic mice with skin-specific overexpression of apelin (K14-Apelin), indicating the protective role of apelin against FFA-induced damage (Sawane et al., 2013).

## Lymphatic Dysfunction Causes Adipose Deposition

### Lymphatic Fluid Causes Adipocyte Maturation and Lipid Accumulation

**FIGURE 1:**
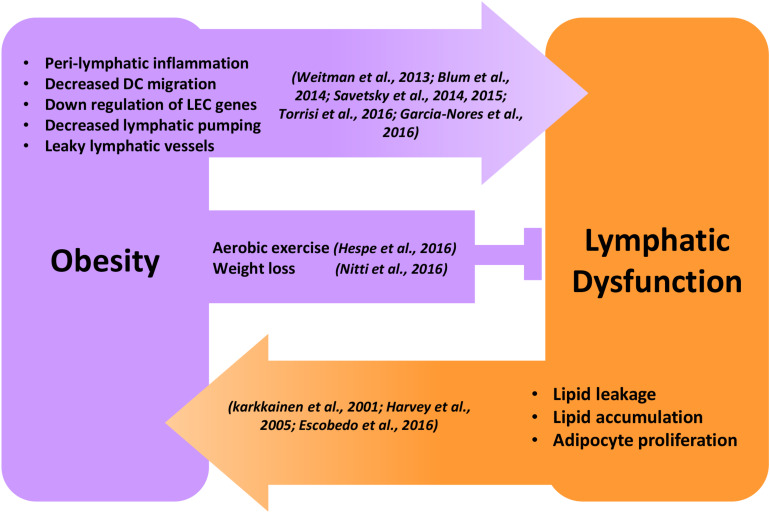
Reciprocal regulation of obesity and lymphatic dysfunction. Schematic illustration showing the reciprocal regulation of obesity and lymphatic dysfunction. Peri-lymphatic inflammation in obesity causes decreased lymphatic pumping, DC migration, LEC gene expression, and increased lymphatic leakage leading to lymphatic dysfunction. Behavioral interventions like aerobic exercise and weight loss inhibit and reverse obesity-induced lymphatic dysfunction. Genetic or surgical lymphatic damage or dysfunction causes malabsorption of lipids, adipocyte proliferation, and progressive accumulation of adipose tissue, leading to obesity.

While it is clear that obesity causes lymphatic dysfunction, it is equally clear that lymphatic dysfunction can cause alterations in adipocyte biology and contribute to local adiposity (Figure 1). Indirect evidence for this hypothesis can be derived from adipose deposition in patients with lymphedema. Lymphedema is a disease that occurs as a result of genetic or acquired abnormalities of the lymphatic system secondary to infections, trauma, or cancer treatment. While early stage lymphedema is characterized by accumulation of interstitial fluid and pitting edema, late stages of the disease result in chronic and progressive adipose tissue deposition, often resulting in massive tissue distortion (Rockson, 2001, 2018; Warren et al., 2007; Mehrara and Greene, 2014). Lipedema, a less well-defined disease resulting in abnormal adipose deposition, has also been shown to be associated with functional alterations in the lymphatic system (Harwood et al., 1996). Lymphatic injury results in expression of IL-6, a broad inflammation marker and an important regulator of adipose tissue homeostasis in lymphedema (Cuzzone et al., 2014). Lymphatic injury in mouse models results in remodeling of adipose tissues and increased expression of adipocyte differentiation genes such as CCAAT/enhancer-binding protein-alpha (CEBP-α), adiponectin, peroxisome proliferator-activated receptor-gamma (PPAR-γ) (Rutkowski et al., 2010; Aschen et al., 2012; Zampell et al., 2012; Tashiro et al., 2017). Other experiments using a rat model in which collecting lymphatics are ligated have shown that insufficient lymphatic drainage results in abnormal lipid accumulation along the vein walls, causing chronic venous insufficiency (Tanaka et al., 2016). These findings are supported by *in vitro* experiments demonstrating that addition of lymphatic fluid (chylous fluid) to culture media of preadipocytes results in cellular differentiation, mature adipocyte gene expression (e.g., PPAR-γ, GLUT-4), and intracellular lipid accumulation (Harvey et al., 2005). More recent studies suggest that FFAs including oleic acid, α-linoleic acid, and palmitic acid are the components of lymphatic fluid that induce adipocyte maturation and differentiation (Escobedo et al., 2016). Taken together, these studies suggest that lymphatic injury causes activation of adipocytes and accumulation of adipose tissues.

### Lymphatics Are Anatomically Co-localized With Adipose Tissues

Anatomic considerations also suggest that the interaction between the lymphatics and adipose tissues is bidirectional. Throughout the body, lymphatic structures are physically located in close proximity to adipose tissues. The open-ended initial dermal lymphatic vessels are located just above the adipose tissue layer in the skin. Major CLVs and trunks are always surrounded by adipose tissues, and lymph nodes are maintained in thick fat pads even in highly lean individuals (Harvey, 2008). These fat pads serve as an energy reservoir that helps to sustain immune responses in lymph nodes (Pond and Mattacks, 1995, 1998, 2002). Surgical removal of lymph nodes postnatally causes failure of fat pad development, suggesting that there is a 2-way communication between adipocytes and the lymphatic system during development (Eberl et al., 2004).

### Congenital Abnormalities of the Lymphatic System Result in Adult-Onset Obesity in Mice

Experiments using transgenic animals with lymphatic defects provide even more direct evidence that lymphatic function can regulate adipose tissue deposition. The homeobox transcription factor Prox-1 is the master regulator of lymphatic specification and identity (Wigle et al., 2002). Prox-1 null mutations are lethal, with death occurring around embryonic day 14.5. However, heterozygous mutations (Prox-1^±^) as well as conditional Prox-1 knockouts survive to adulthood despite anatomic and functional lymphatic abnormalities. These mice have leaky lymphatics and chylous ascites that partially resolve (Harvey et al., 2005). Interestingly, mice with heterozygous Prox-1 mutations become obese as adults (by 4 months of age) even when fed a normal chow diet. These mice display no differences in food intake but nevertheless gain significantly more weight, accumulate adipose tissues in both intra-abdominal and subcutaneous tissues, and develop metabolic abnormalities, including insulin resistance when compared with littermate controls. Restoration of lymphatic Prox-1 signaling not only rescues lymphatic function but also prevents abnormal adult-onset weight gain and metabolic abnormalities (Escobedo et al., 2016). Interestingly, although the most common clinical cause of obesity is over-nutrition rather than genetic abnormalities, recent genome-wide association studies have identified single-nucleotide polymorphisms in the Prox-1 that are linked with increased waist circumferences and obesity (Kim et al., 2013; Lecompte et al., 2013). However, more in-depth research is warranted attributing these results to lymphatic phenotype as Prox-1 is also expressed by liver and skeletal muscle which significantly contributes to metabolism and energy homeostasis.

Other mouse models with genetic abnormalities of the lymphatic system that cause lymphatic dysfunction also display abnormal adipose deposition. For example, mice with heterozygous inactivating mutations in Vegfr-3 (also known as Chy mice) develop chylous ascites post-natally that resolve spontaneously. However, these mice develop swollen feet and increased subcutaneous adipose tissue deposition as compared to wild-type mice (Karkkainen et al., 2001; Dellinger et al., 2007; Rutkowski et al., 2010). Similar findings have been reported in angiopoietin-2 (Ang-2) knockout mice. These mice have abnormal embryonic lymphatic patterning and develop adult-onset foot pad swelling due to adipose deposition (Dellinger et al., 2008).

## Regulation of Metabolic Abnormalities by the Lymphatic System

Lymphatic vessel function plays a key role in regulation of wide variety of biological phenomenon, and, as a result, lymphatic abnormalities are associated with many pathologic conditions, including cancer, autoimmune disorders, transplant rejection, atherosclerosis, diabetes, myocardial infarction (MI), and hypertension, to name a few. However, in the context of this review, we will briefly discuss those pathologies that are concerned with lymphatic function and metabolic disorders.

### Lymphatic Function in Hypercholesterolemia and MI

Obesity is frequently associated with hypercholesterolemia. However, many obese patients exhibit normal blood lipids, and not all hypercholesterolemia patients are obese (Miettinen, 1971; Guerrero-Romero and Rodriguez-Moran, 2006). These irregularities indicate that some other common factor might play a key role in hypercholesterolemia pathology. Accumulation of lipids in the extracellular tissues is the key feature of hypercholesterolemia. Recent studies have shown that the lymphatic system plays an important role in cholesterol metabolism by transporting cholesterol from the peripheral tissues to the liver via a process called RCT (Lim et al., 2013; Martel et al., 2013; Veronique and Ying, 2013; Huang et al., 2015). Studies using Chy mice or surgical ligation of lymphatic channels results in decreased RCT and cholesterol clearance from the tissues (Lim et al., 2013; Martel et al., 2013). The efflux of cholesterol from peripheral tissues to systemic circulation through lymphatic vessels is not passive, but an active process could dependent on uptake and transcytosis of HDL by scavenger receptor class B type 1 (SR-B1) expressed on LECs (Lim et al., 2013). This concept is supported by the fact that patients with primary lymphedema often display xanthomas (cholesterol-rich deposits in the skin) (Berger et al., 1972; Romani et al., 2012). Poor lymphatic function in primary lymphedema causing increased cholesterol accumulation might contribute to fat deposition, a major symptom of lymphedema. Impaired lymphatic function is also linked to lipoprotein metabolism, increased plasma cholesterol levels, and enhanced atherogenesis (Vuorio et al., 2014). Other studies using apolipoprotein E knockout (ApoE^–/–^), an important protein in fat metabolism, or low-density lipoprotein receptor knockout (Ldlr^–/–^) models of hypercholesterolemia have shown that these mice also have severe lymphatic dysfunction, suggesting the interaction between elevated cholesterol levels and lymphatic function may be bidirectional (Lim et al., 2009; Milasan et al., 2019). Based on these findings, RCT by lymphatic vessels from the arterial wall or atherosclerotic plaques has been discussed as a potential anti-atherogenic therapeutic target in patients with cardiovascular diseases (Milasan et al., 2015; Zheng et al., 2018; Csanyi and Singla, 2019).

The most common complication of obesity and atherosclerosis is MI (Zhu et al., 2014). It is caused by obstruction of coronary circulation, leading to cardiomyocyte loss and scar tissue accumulation culminating in heart failure. The primary event post MI is a potent innate inflammatory reaction for phagocytosis of the dead cell debris and clearance. Reports indicate that after an MI incident, there is a profound remodeling of cardiac lymphatic vessels, and that augmentation of this response by VEGF-C treatment can lead to improved cardiac function (Klotz et al., 2015). These findings are also supported by data from soluble VEGFR-3 decoy transgenic mice (sVEGFR-3) and Chy mice, which reveal that downregulation of VEGFR-3 signaling structurally alters the cardiac lymphatic vasculature and increases mortality after MI (Vuorio et al., 2018). In addition to VEGF-C, adrenomedullin, a known cardioprotective peptide, is also shown to induce cardiac lymphatic growth and function after MI (Trincot et al., 2019). Mechanistically, after MI, cardiac lymphatic vessels clear acute inflammatory immune response, help in resolution of cardiac inflammation, and inhibit scaring and fibrosis and this process is dependent on cardiac lymphatic vessel expression of LYVE-1 (Vieira et al., 2018). Taken together, improving both cardiac lymphatic regeneration and function has the promise to be a therapeutic strategy for infracted myocardium recovery.

### Lymphatic Function and Diabetes

Obesity is highly associated with diabetes and chronic hyperglycemia (Al-Goblan et al., 2014). The role of lymphatic function on diabetes, and how hyperglycemia regulates lymphatic vessels, is still an active topic of investigation. Impaired wound healing is a severe complication of diabetes, and lymphatic regeneration is an integral part of wound healing; therefore, it is imperative to understand how lymphatic function influences diabetes and vice versa (Saaristo et al., 2006; Brem and Tomic-Canic, 2007; Clavin et al., 2008). Using a mouse corneal suture model, it is reported that lymphangiogenesis is significantly suppressed in a diabetic mouse model of leptin receptor deficiency (db/db). These mice also showed impaired wound healing due to defective lymphatic vessel proliferation and collateral lymphatic vessel formation (Maruyama et al., 2007). Uptake of dextran tracer from peripheral tissues to lymph nodes was decreased in alloxan-induced diabetic rats, and insulin treatment rescued this phenomenon, indicating possible decreased lymphatic function in diabetes (Moriguchi et al., 2005). Other studies analyzing gene expression pathways in LECs isolated from patients with diabetes have shown that pro-inflammatory, pro-lymphangiogenic, and lipid-shuttling gene expression pathways are increased in diabetes, while genes related to immune defense, apoptosis mediation, and small-compound transporters are downregulated (Haemmerle et al., 2013). Using cultured LECs, Lee et al. showed that prolonged hyperglycemia induced insulin resistance and these LECs showed disruption of adherent junction proteins, indicating negative effects of hyperglycemia on LECs (Lee et al., 2018). Using lymphatic muscle cells (LMCs), the same group found that hyperglycemia strongly inhibits LMC contractile function by alteration of cellular bioenergetics and activation of inflammatory signaling in lymphatic muscle. These results were supported by an elegant *ex vivo* study by Scallan et al. (2015) that used db/db diabetic mice to study CLV function. They found that collecting lymphatic vascular integrity is disrupted in Type 2 diabetes due to low NO bioavailability. Scallan et al. (2015) further showed that inhibition of the phosphodiesterase 3 (PDE3) enzyme, that is normally inhibited by NO signaling, restored lymphatic vascular integrity and improved lymphatic function (Alexander and Becker, 2015; Scallan et al., 2015). Taken together, these findings indicate that diabetes impairs lymphangiogenesis, lymphatic permeability, and contractile function. However, the molecular events leading to impaired lymphangiogenesis during diabetes were unknown known recently. Using a streptozotocin-induced model of diabetes in mice fed an HFD, Wu et al. (2018) found that lymphangiogenesis and lymphatic function were impaired in diabetes due to downregulation of VEGFR-3 expression on LECs. This VEGFR-3 downregulation is mediated by epsin-mediated endocytosis (Wu et al., 2018). The specific deletion of epsin in LECs not only improved lymphatic growth but also enhanced lymphatic function in HFD-fed diabetic mice. These findings reveal that diabetes-induced lymphatic dysfunction is mainly mediated by endocytosis of VEGFR-3 by epsins and depletion of epsins in a potential therapeutic target to improve lymphatic function in diabetes.

### Intestinal Lacteal Vascular Endothelial-Cadherin (VE-Cadherin) Zippering

Lymphatic leakiness causes chylus leakage and obesity. Lacteals are the intestinal villi lymphatic vessels with button-like VE-cadherin junctions. Vascular factors such as VEGF-C, angiopoietin-2, and DLL4 are reported to maintain VE-cadherin junctional integrity and regeneration of lacteals (Zheng et al., 2014; Bernier-Latmani et al., 2015; Suh et al., 2019). These VE-cadherin intercellular button-like junctions allow fluid and chylomicrons into lacteals and, eventually, into blood circulation (Cifarelli and Eichmann, 2019; Hokkanen et al., 2019). Converting the lacteal LEC button junctions to zipper-like junctions prevents chylomicron entry into lacteals thus reducing lipid absorption and protection against diet-induced obesity. A study by Zhang et al. (2018) reported that endothelial-specific genetic deletion of Neurophilin-1 (Nrp-1) and vascular endothelial growth factor receptor 1 (Vegfr-1) induced lacteal VE-cadherin junction zippering and chylomicron malabsorption. Deletion of Nrp-1 and Vegfr-1 on endothelial cells increased signaling through VEGFR-2 due to increased VEGF-A bioavailability, causing zippering of the lacteal junctions. This resulted in malabsorption of the chylomicrons in these transgenic mice and turning them resistant to diet-induced obesity. As an alternative pharmacologic approach, Rho-associated protein kinase (ROCK) inhibitor Y27632 was used to induce straightening lacteal junctions, causing reduced chylomicron absorption into lacteals of wild-type mice. This novel intervention of closing lymphatic junctions by transformation of buttons to zippers is quite intriguing. However, detailed research is needed to determine the adverse effects of lacteal zippering on overall intestinal health.

## Future Directions

Although considerable progress has been made in understanding the reciprocal relationship between obesity and lymphatic function, many questions still remain. One of the major topics that requires addressing is identification of the mechanisms by which obesity injures the lymphatic system. While it is clear that inflammation plays a key role in this process, the cellular pathways that mediate lymphatic dysfunction in obesity, both clinically and in mouse models, remain largely unknown. Equally important in this discussion are studies analyzing the reversibility of these changes clinically. Thus, while preclinical models indicate that anti-inflammatory medications and behavioral modifications can reverse the pathologic effects of obesity on the lymphatic system, it remains to be seen whether these abnormalities can be reversed in patients with longstanding obesity. Understanding the cellular mechanisms that regulate lymphatic function in obesity and understanding how these effects are normalized with targeted treatments, will have a profound impact on a variety of metabolic diseases, and is an urgent biomedical need.

## Author Contributions

RK, BM, HP, JB, and CL performed the literature search, compiled and wrote the manuscript. HP, JB, and JS proofed the manuscript. RK and HP created the figures. All authors contributed to manuscript revision and read and approved the submitted version.

## Conflict of Interest

The authors declare that the research was conducted in the absence of any commercial or financial relationships that could be construed as a potential conflict of interest.
